# State-of-the-Art Technologies for Understanding Brassinosteroid Signaling Networks

**DOI:** 10.3390/ijms21218179

**Published:** 2020-10-31

**Authors:** Haijiao Wang, Song Song, Huaqiang Cheng, Yan-Wen Tan

**Affiliations:** 1 State Key Laboratory of Crop Stress Adaptation and Improvement, School of Life Sciences, Henan University, Kaifeng 475001, China; wanghaijiao@henu.edu.cn; 2 Department of Basic Courses, Zhejiang University of Water Resources and Electric Power, Hangzhou 310018, China; songs@zjweu.edu.cn; 3 State Key Laboratory of Surface Physics, Multiscale Research Institute of Complex Systems, Department of Physics, Fudan University, Shanghai 200433, China; huaqiangch@gmail.com

**Keywords:** technologies, brassinosteroids, signaling, proximity labeling, single-molecule methods

## Abstract

Brassinosteroids, the steroid hormones of plants, control physiological and developmental processes through its signaling pathway. The major brassinosteroid signaling network components, from the receptor to transcription factors, have been identified in the past two decades. The development of biotechnologies has driven the identification of novel brassinosteroid signaling components, even revealing several crosstalks between brassinosteroid and other plant signaling pathways. Herein, we would like to summarize the identification and improvement of several representative brassinosteroid signaling components through the development of new technologies, including brassinosteroid-insensitive 1 (BRI1), BRI1-associated kinase 1 (BAK1), BR-insensitive 2 (BIN2), BRI1 kinase inhibitor 1 (BKI1), BRI1-suppressor 1 (BSU1), BR signaling kinases (BSKs), BRI1 ethyl methanesulfonate suppressor 1 (BES1), and brassinazole resistant 1 (BZR1). Furthermore, improvement of BR signaling knowledge, such as the function of BKI1, BES1 and its homologous through clustered regularly interspaced short palindromic repeats (CRISPR), the regulation of BIN2 through single-molecule methods, and the new in vivo interactors of BIN2 identified by proximity labeling are described. Among these technologies, recent advanced methods proximity labeling and single-molecule methods will be reviewed in detail to provide insights to brassinosteroid and other phytohormone signaling pathway studies.

## 1. Introduction

**Figure 1:**
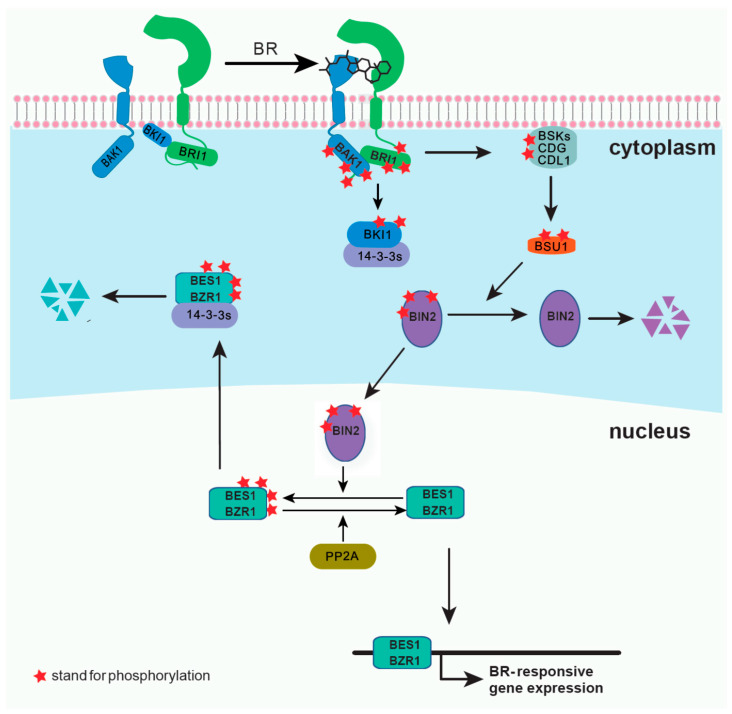
A simplified model for brassinosteroids (BRs) signaling pathway.

Brassinosteroids (BRs) are plant steroid hormones critical in numerous developmental and physiological processes, such as stem elongation, vascular differentiation, seed size, male fertility, flowering time, senescence, cell division, and resistance to biotic and abiotic stresses [1,2]. Because of the discovery of the BR receptor BR-insensitive 1 (BRI1) [3,4], several key components of the BR signaling pathway have been reported (Figure 1). BRI1 is a cell surface-localized receptor kinase [4,5]. Without BRs, BRI1 is maintained at an inactive state by its C-terminal autoinhibition [6], and by a negative regulator, BRI1 kinase inhibitor 1 (BKI1), which prevents the interaction of BRI1 with its coreceptor BRI1-associated kinase 1 (BAK1) [7,8]. In the downstream signaling pathway, two main transcription factors, BRI1 ethyl methanesulfonate (EMS) suppressor 1 (BES1) and brassinazole resistant 1 (BZR1), are phosphorylated by BR-insensitive 2 (BIN2), and some members of 14-3-3 proteins can bind and retain the phosphorylated BES1 and BZR1 in the cytoplasm [9,10,11,12]. BR binding to the island domain of BRI1 induces a conformational change of the ectodomain [13,14], which activates the intracellular domain, releases the autoinhibition of BRI1 C-terminus, and phosphorylates BKI1 [6,8,15]. The phosphorylated BKI1 dissociates from the plasma membrane to interact with 14-3-3λ and κ, and competitively inhibits the binding of 14-3-3λ and κ with BES1/BZR1, leading to BR-regulated gene expression [15]. At the same time, the activated BRI1 interacts with and transphosphorylates its coreceptor BAK1 to recruit positive regulators, such as BR signaling kinases (BSKs) and constitutive differential growth 1 (CDG1), and phosphorylates them [16,17]. Phosphorylated BSKs and CDG1 then activate phosphatase BRI1-suppressor 1 (BSU1) and its homologs BSU1 like (BSLs) to inactivate BIN2 [16,17]. Subsequently, protein phosphatase 2A (PP2A) dephosphorylates BES1/BZR1, leading to the expression of the BR-responsive genes [18,19]. Several reviews have already summarized the most recent discoveries in the BR signaling pathway [2,20]. In this review, we introduce several new technologies and methodologies that have been applied to the study of BR signal transduction and have led to discoveries unattainable by conventional methods. Although our focus is on the BR pathway, the applications of each method can provide inspirations for studies on other plant signaling pathways.

The development of the understanding of the BR signaling pathway has progressed with the advancements in biotechnology. Molecular genetics provides the fundamentals for the study of signaling pathways. In particular, the combination of forward and reverse genetics facilitated the identification of BR signaling components. Here, we summarize traditional and new methods used. Conventional methods, including EMS mutant screening, activation tagging, yeast two-hybrid (Y2H) screening, two-dimensional (2D) difference gel electrophoresis (DIGE), and liquid chromatography (LC)–tandem mass spectrometry (MS/MS), the applications of which have laid the foundation of our current understanding of the BR signaling pathway. New technologies, such as bioinformatics, clustered regularly interspaced short palindromic repeats (CRISPR) system, proximity labeling (PL), and single-molecule technology, have considerably enhanced this understanding. In this review, we illustrate how these technologies have been used in BR network studies, with emphases on PL and single-molecule technology. We review the development of these two methods, highlighting their application in planta. The coverage of these methods can help researchers in the field gain new insights for their own studies.

## 2. Identification of New Components of BR Signaling

### 2.1. Forward Genetics

Forward genetics is the screening of phenotypes of interest under different backgrounds and conditions to identify the gene that causes a certain phenotype. It laid the foundation for our understanding of the BR signaling pathway in the early days. The application of forward genetic screening usually begins with naturally or artificially generated mutations.

#### 2.1.1. Screening of EMS Mutants and Identification of BRI1, BIN2, and the Transcription Factors BES1 and BZR1

The first identified component of the BR signaling pathway is BRI1, the receptor of BR. In 1996, Clouse et al. used an EMS population to screen BR-insensitive mutants on 10^−6^ or 10^−7^ M final concentration of eBL, the synthetic most active brassinolide [3]. EMS is a monofunctional ethylating chemical agent that can cause gene mutation or deletion in various genetic test systems from viruses to mammals. In the screening, a mutant, did not respond to eBL in hypocotyl elongation and primary root inhibition assays, was identified and termed *bri*1. In 1997, 18 *Arabidopsis* dwarf mutants unable to respond to exogenously added eBL were identified [4]. These mutants were cloned and were found to encode BRI1, a leucine-rich receptor-like kinase. BIN2 was also screened using the EMS population by analyzing dwarf and semidwarf mutants by different labs [4,21,22,23]. BRI1 and BIN2 were obtained by screening eBL-insensitive mutants; however, the transcription factor BZR1 was isolated from EMS populations, which did not respond to brassinazole (BRZ), a triazole compound that specifically blocks brassinolide biosynthesis [11]. By adding BRZ in the medium, a dominant mutant with a long hypocotyl in the dark was isolated and named *bzr1-1 Dominant* (*bzr1-1D*). BES1, a BZR1 homolog, was also identified using EMS but under different conditions. When researchers screened the suppressors of *bri1-119*, a weak allele of *bri1* that harbored a missense mutation in the 70-amino-acid island domain and that completely suppressed *bri1-119* phenotypes, long and bending leaf petioles, was identified and named as *bes1*. EMS screening is highly efficient and can identify major genes in a signal, but it cannot identify homologous genes playing redundant functions.

#### 2.1.2. Activation Tagging

*Agrobacterium*-mediated transformation is a vital tool for basic plant research. Transfer DNA (T-DNA) integration into the plant genome can cause loss-of-function mutation. Activation tagging using T-DNA inserts can generate random genomic insertions containing strong transcriptional enhancers that can increase the expression of nearby genes [24]. To identify the downstream components of BRI1, an activation tagging transgenic population in *bri1-5,* which contains a point mutation resulting in a C69Y amino acid substitution in the extracellular domain of BRI1, was constructed, and its suppressors were screened. Both BAK1, the BRI1 coreceptor, and BSU1, a BIN2 phosphatase, were identified to suppress *bri1-5* [25,26]. Li et al. identified a mutant that partially suppressed the phenotype of *bri1-5* and named it *bak1-1D* [25]. By mapping, *bak1-1D* phenotypes were found caused by an insertion of activation tagging T-DNA 618 bp upstream of the start codon of BAK1, leading to approximately 30 times higher BAK1 mRNA levels. Chory’s group found a suppressor that can partially rescue *bri1-5* phenotypes and designated it as *bsu1* [26]. Its phenotype was caused by activation tagging at its downstream. *cdg1-D* indicated a twisted or spiral hypocotyl and an open-hook structure in the dark and excessively epinastic leaves in the light; it was also isolated with activation tagging and was involved in BR signaling [27]. By 2011, the roles of CDG1 in BR signaling were clarified: CDG1 is a BRI1 substrate and a BSU1 kinase [28]. Activation tagging can identify genes with homologs but with low efficiency, so most redundant genes cannot be obtained using this method.

### 2.2. Reverse Genetics

In contrast to forward genetics, reverse genetics starts from molecular engineering of a gene to search for alterations of phenotypes. Genome sequencing enables easy characterization of the gene sequence. Several popular methods directly identify signaling components: Y2H, two-dimensional polyacrylamide gel electrophoresis, LC–MS/MS, bioinformatic prediction, and CRISPR.

#### 2.2.1. Y2H

The Y2H system was developed by Fields and Song to detect protein–protein interactions (PPIs) [29]. This system is based on the phenomenon that the DNA-binding domain (BD) and activation domain (AD) of eukaryotic transcription factors can function separately. When BD and AD are in close proximity, gene expression can be activated by the transcription factor (please see Paiano et al. (2018) for details) [30]. The BRI1 coreceptor BAK1 was also identified by screening BRI1 interacting proteins through Y2H [31]. In addition, transthyretin-like protein (TTL) and BKI1, two negative regulators of BR signaling, are also screened by Y2H with the kinase domain of BRI1 as a bait and a cDNA library from *Arabidopsis* apical meristem [7,32]. In addition, Y2H can detect proteins that directly interact with signaling components, and the interacting protein identified may be the component of the signaling pathway or crosstalk protein with another signaling pathway. However, false-positive clones often appear in Y2H, and both quality and origin of the cDNA library are crucial to obtain desirable results with Y2H.

#### 2.2.2. 2D-DIGE

Polyacrylamide gel electrophoresis (PAGE) is a method for protein separation (for details, please read Meleady (2018)) [33]. Two-dimensional difference gel electrophoresis (2D-DIGE) is a modified version of 2D-PAGE. In 2D-DIGE, different protein samples are labeled with different fluorescent tags [33]. To screen new components of BR signaling, seedlings of *det2-1*, a BR synthesis mutant, were treated with eBL (the most active form of BRs) or mock. By screening BR-induced proteins through 2D-DIGE, which displayed an increase in acidic forms and decrease in basic forms, BSK1 and BSK2 were identified [16]. When searching for the homologs of BSK1 and BSK2, BSK3 and BSK5 were identified and found to play a similar role in BR signaling. BSKs are BRI1 substrates and phosphorylate BSU1 to enhance the phosphatase of BSU1. 2D-PAGE and 2D-DIGE can identify quantitative differentially expressed proteins under different experimental conditions. However, they cannot identify low abundant proteins, proteins with extreme pI, and proteins with similar isoelectric and molecular weight. Furthermore, analysis of 2D-PAGE and 2D-DIGE data is also challenging.

#### 2.2.3. LC–MS/MS

LC–MS/MS-based proteomics is a powerful tool for identifying and quantifying proteins. LC–MS/MS can identify as many as several thousands of peptide sequences, including proteins with low expression levels. BKI1 is a negative regulator of BRI1. When BRs bind to receptor BRI1, BKI1 is dissociation from the plasma membrane. However, its dissociation mechanism and its function in the cytosol were unknown. To explore the interacting protein of BKI1, BKI1-FLAG was immunoprecipitated by ANTI-FLAG M2 affinity gel from *BKI1-FLAG* overexpression plants and the enriched proteins were analyzed through LC–MS/MS. For BKI1, several 14-3-3 proteins were found in the list of interactor proteins with 90% confidence. 14-3-3 proteins were further proved to interact with phosphorylated BKI1 and help in BKI1 dissociation [15]. Through LC–MS/MS, several key proteins that link the interaction of BR signal components with other signals were identified, such as BIN2 interacting with SnRK2.2/2.3/2.6 to activate ABA signaling [34]. However, LC–MS/MS is a global proteomic tool that can identify indirectly interacting proteins.

#### 2.2.4. Bioinformatics for Identification of BR Signaling Components

The use of bioinformatics approaches is increasing in biological research. Bioinformatics can process multiomics data and predict all types of modifications, and thus, it has been used considerably. In BR signaling, bioinformatics has been used to analyze gene expression under different conditions or in different tissues, evaluate mutants by RNA-sequencing, illuminate the crosstalk between BR and other signals [34,35,36,37], and predict protein modification and transcript, such as a new BES1 transcript. BES1 was reported as the transcription factor of BR signaling in 2002; however, its overexpression did not generate any BR-related phenotypes. Jiang et al. used bioinformatics to reveal an alternative transcription start site in BES1 promoter, leading to a longer transcript than the reported BES1—designated as BES1-L [38]. BES1-L overexpression causes an obvious BR-related plant growth phenotype. BES1-L interacts with BES1-S (the short transcript) to help the nuclear localization of BES1-S to enable DNA binding and regulation of hundreds of BR-responsive genes. As data mining develops, bioinformatics will be an indispensable tool in biological research.

#### 2.2.5. CRISPR/Cas9 System

The CRISPR system functions as part of an adaptive prokaryotic immune system [39]. In 2012, CRISPR was developed into a genomic tool and has since revolutionized the field of molecular biology. The most prevalent system is the CRISPR/Cas9 system, which was modified from the type II CRISPR system [40]. CRISPR/cas9 can confirm the function of a gene that has no loss-of-function mutants in a signaling pathway. In BR signaling, BKI1 can interact with BRI1 and prevent its interaction with its coreceptor BAK1. However, BKI1 has six homologous genes in *Arabidopsis*, including membrane associated kinase regulator 1 (MAKR1), which can also interact with BRI1 [41]. Jiang et al. examined whether the function of BKI1 and MAKR1 is redundant by disrupting the C-terminal region of BKI1 using CRISPR/Cas9; they found that the genes were not redundant in BR signaling [42]. In addition, the T-DNA mutants of BES1 and its homologous BZR1 have no BR-related plant growth phenotypes. BES1 has five homologous genes in *Arabidopsis*: BZR1, BES1/BZR1 homolog 1 (BEH1), BEH2, BEH3, and BEH4. However, the traditional methods of T-DNA insertion or EMS mutation cannot obtain the loss-of-function mutants containing all the BES1 homologous genes. The BES1 hextuple mutants (BES1-h) were generated by CRISPR combined with T-DNA insertion [43]. BES1-h exhibited a similar phenotype to BRI1 null mutant, indicating that BES1 and its homologs are the transcription factors of BR signaling and redundantly regulate the expression of BR-responsive genes. Through analyzing the BES1-h mutants, BES1 and its homologs were also found to redundantly regulate tapetum development downstream of EMS1-TPD1-SERK1/2 signaling [43,44]. Furthermore, disrupting the upstream open reading frame of BRI1 using CRISPR/Cas9 can increase the translation of BRI1 and enhance BR signal output [45], indicating this technique can play a vital role in signaling regulation.

## 3. Examination of the Dynamic Regulation Mechanism of BR Signaling Components

After the main components of BR signaling are identified, the regulation mechanism between them is examined, including interactions, structure analysis, phosphorylation, acetylation, and polyubiquitination. However, these mechanisms are in a quiescent condition. Proximity labeling (PL) is a powerful new technique for studying protein–protein interactions (PPIs). In recent years, the dynamic interaction has also been examined using single-molecule technology.

### 3.1. Plant Hormone Signaling Pathway Studied Using PL

PPIs are the basic elements of signaling pathways. Currently, the gold standards for studying PPIs are Y2H, co-immunoprecipitation, pulldown assays, and in vivo Förster-type resonance energy transfer (FRET) or bimolecular fluorescence complementation (BiFC) assays. However, each of these has its own limitations. Y2H, co-immunoprecipitation, and pulldown assays can be used to explore potential unknown interaction partners of a bait protein. However, they usually suffer from false negatives and false positives. Furthermore, they fail to detect interactions that are weak or appear only transiently. Moreover, for membrane lipid-assisted PPIs, the trace of interactions can easily be lost during the harsh lysing and affinity pulldown processes. In vivo FRET or BiFC assays can, in principle, capture transient PPIs, but these methods are in the binary candidate format and require fluorescent labeling. Therefore, the successful execution of the assays depends on the transfection efficiency, and the assays are prone to disruption by the native autofluorescence background of live cells, especially plant cells. PL has been a perfect complement for the conventional PPI assays. PL uses engineered ligase or peroxidase to cast specific tags, usually biotin, on certain residues on proteins appearing in the proximity of the original bait protein. The interactome information can be analyzed from the cell lysate with affinity chromatography–MS/MS.

#### 3.1.1. PL Can Be Categorized by the Enzyme Used

**Figure 2:**
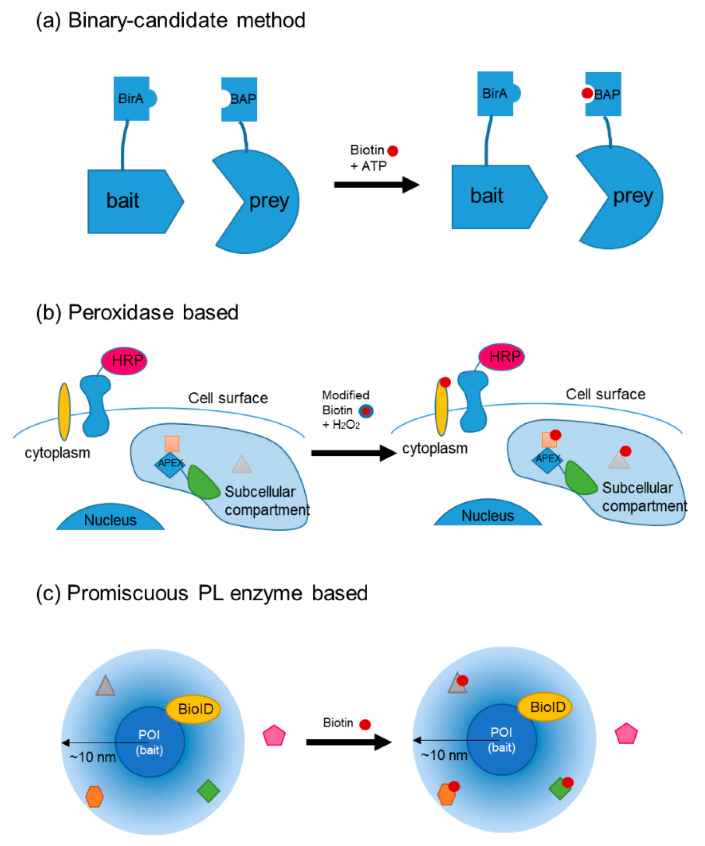
Three major categories of proximity labeling. Each category is illustrated by one or two representative enzyme systems. (**a**) Binary-candidate PL method using BirA and BAP attached to the bait and prey proteins, respectively. The reaction is induced by biotin and ATP; (**b**) Peroxidase based PL using Horseradish peroxidase (HRP)-based method on cell surface or APEX in a cellular compartment. The reaction is induced by modified biotin molecules and H_2_O_2_; (**c**) Promiscuous PL enzyme illustrated by BioID, which has a working distance around 10 nm. This PL reaction is induced by biotin.

Depending on the enzymes used, the functional strategies and applications of PL can fall into several different categories. Many PL systems originate from the engineering of a well-studied biotin ligase BirA from *Escherichia coli*. BirA catalyzes the reaction of synthesizing biotinoyl-AMP (bio-5′-AMP, or bioAMP) from biotin and ATP. The enzyme sequesters bioAMP at its active site and transfers it to a specific peptide nearby. This proximity-dependent reaction provides the basic blueprint for PL enzymatic systems. The more specific BirA recognizes a biotin acceptor peptide (BAP). This BirA/BAP pair has been designed as a binary-candidate interaction detection pair [46]. It can be fused to the protein of interest (POI) and potential interaction partners, and their interactions can be examined in relevant in vivo conditions (Figure 2a). Compared with common BiFC assays, PL is more sensitive and free form background autofluorescence. The traces or histories of PPI are stored and can be detected later. Nonetheless, the detection is completed via LC–MS/MS or staining on fixed cells, so the dynamics and temporal information of PPIs are lost in the process. Several improved variations of BirA/BAP-based binary-candidate methods exist, including biotin labeling of intercellular contacts (BLINC), interaction-dependent probe incorporation mediated by enzymes (ID-PRIME) [47], and specific protein association toll giving transcriptional readout with rapid kinetics (SPARK) [48]. We have made a figure (Figure 2) and a table (Table 1) showing the nature and applications of these enzymatic probes.

Another type of PL method is based on peroxidases instead of biotin ligases (Figure 2b). Peroxidases can generate reactive free radicals, which can be covalently bind to certain amino acids, such as tryptophan and tyrosine. The nature of free radical reactions accelerates the labeling process, and these reactions appear within a very short working distance, usually tens of nanometers. Various methods exist depending on the peroxidase system used, such as enzyme-mediated activation of radical source (EMARS) [49,50], selective proteomic PL using tyramide (SPPLAT) [51], engineered ascorbate peroxidase (APEX), and APEX2. The applications of horseradish peroxidase (HRP)-based methods are limited to the cell surfaces since they require an HRP-linked antibody, which is a large complex, to attach to the POI. APEX and APEX2 were engineered from plant ascorbate peroxidase. Originally developed for staining under electron microscopy [52], APEX was designed to be genetically fused to any POI. Unlike the reaction catalyzed by HRP, APEX works on radicals with a half-life three orders of magnitude shorter than that of HRP and therefore a shorter labeling radius. It can be targeted to different cellular compartments in live cells. APEX facilitates the study of various subcellular locations without harsh organelle purification processes.

#### 3.1.2. Search for the PL Method Suitable for In Planta Studies

The aforementioned strategies for enzyme-catalyzed PL have not been widely applied in planta. For the binary-candidate methods, the most well-established approaches are sufficient for light-insensitive systems. The highly reactive peroxidase-based PL systems require hydrogen peroxide (H_2_O_2_) to complete the reaction. Hydrogen peroxide is also a plant signaling molecule and is toxic to live cells under high concentrations. Another category of enzyme-catalyzed PL uses an engineered mutant the biotin ligase BirA, which works promiscuously instead of in a binary candidate format [61]. The mutant enzyme sequesters the bioAMP at its active site, albeit not as strongly as the wild type, and transfers it to a nearby amine (lysine sidechain). The working distance is estimated to be approximately 10 nm [62], enabling PL detection without BAP implementation. Therefore, proteomic screening can be performed in physiologically relevant live cells. Because the reaction requires only biotin supplements, it is of low toxicity to plant cells. This method, known as proximity-dependent biotin identification (BioID) (Figure 2c) [55], is suitable for eukaryotic cells because the prokaryotic biotin ligase BirA is orthogonal to eukaryotic cells.

BioID has been widely used to study PPIs. Although false negatives may occur if the interaction partner does not have exposed lysine sidechains, BioID can preserve the trace of weak and transient PPIs. BioID2 is an improved version of BioID with a reduced size from 321 to 233 residues [56]. It is designed by trimming the BioID enzyme down to only the active domain. The application of the BioID strategy involves fusing the promiscuous BirA mutant (denoted as BirA* subsequently) to the POI and induces the expression of the fusion construct with biotin supplementation. The incubation time is usually 15–18 h at 37 °C. At temperatures <37 °C, the reactivity decreases considerably. This may prevent the applications in certain cell lines. To overcome such hurdles, TurboID was developed by directed evolution to screen for highly reactive BioID variants [57]. After reforming the enzyme, TurboID can reduce the incubation time from 15 h to 10 min and maintain catalytic efficiency at <37 °C. Such characteristics make TurboID a potent probe for PL applications.

#### 3.1.3. Early PL Applications in Planta

BioID was first used in planta to create the physiologically relevant proteomics for the rice genes OsFD1 and OsFD2 [63]. In this work, the promiscuous BioID enzyme, BirA*, was modified to adapt to plant protoplast. The modification involved removing a cryptic intron from BirA* because alternative splicing of BirA* occurred in rice cells. The modified enzyme was named BirAG. In rice protoplast, BirAG-induced biotin ligation worked best with a supplement of 50 µM biotin and an incubation time of 24–48 h. The mass spectrometry (MS) results from the OsFD2 biotin PPI screening generated 135 proteins. After eliminating backgrounds and controls, 12 putative OsFD2 interaction partners were identified through PL screening. Some of these proteins were 14-3-3-related proteins, which are known OsFD2 interacting factors. Finally, three newly detected PPI partners generated positive BiFC results, among which two were mainly located in the nucleus. Immediately after its application in rice, BioID was applied to the whole plant in the model system *Arabidopsis thaliana* [64]. The authors used a HopF2-BirA* transgenic plant to survey the interactome of HopF2 via PL. Notably, the PL assay did not capture BAK1, the known interaction partner. The authors attribute this to the limited distance range of the BioID method (approximately 10 nm) or potentially a lack of accessible primary amines on the BAK1 surface.

The advent of TurboID promoted research in plant signaling systems. For effector-triggered immunity in plants, nucleotide-binding leucine-rich repeats (NLRs) are at the frontline of the defense signaling pathways. However, the early signaling partners or adaptors of NLRs are very difficult to identify [65], partly because NLRs appear in low copy numbers in cells. To overcome this obstacle, Zhang et al. used a TurboID-based PL assay to study the interactome of N NLR during infection with the tobacco mosaic virus (TMV) [66]. In a preliminary investigation involving N NLR’s known interaction partner, Hsp90, the authors found that TurboID outperformed BioID not only in reaction efficiency but also in specificity. However, the bottleneck for this reaction is the activation of the N-mediated defense response, necessitating a 12-h incubation time before harvesting for LC–MS/MS. PL of N NLR combined with isobaric tagging through MS/MS enabled quantitative evaluation of enrichment factors. Approximately 100–200 proteins were identified with or without p50 effector. Ten of the genes covering a range of enrichment factors were chosen for further investigations. The gene silencing experiments on *Nicotiana benthamiana* plants showed that 6 of 10 knockdown plants collapsed after inoculation with TMV. Notably, *NbUBR7* was found to be a negative regulator of N, and it interacted with the toll/interleukin-1 receptor (TIR) domain of N. UBR7 is a highly conserved E3 ubiquitin ligase and part of the ubiquitin–proteasome system. It was not found in the p50 expression PL group, and further experiments indicated that p50 may disrupt the interaction between UBR7 and N. These findings provide clues for how the cells maintain a low level of N protein and a model for early activation events.

#### 3.1.4. TurboID for BR Signaling Pathway

Besides elucidating the activation mechanism of small amounts of plant immune receptors, PL-based PPI proteomics assay is a powerful tool to study signaling pathways. Because signal transduction is usually a dynamic relay between signaling network members, it is transient by nature. The BR signaling pathway was investigated through TurboID-mediated PL mapping of BIN2 proteomics [67], and 280 proteins were identified in the BIN2-YFP-TbID run but not in the control set. Two known BIN2 interactors that were not captured by previous pulldown experiments were detected in this pool of proximal proteins. Some of the known BIN2 interactors were not detected through PL–MS, such as BIN2′s E3 ligase KIB1, which may be attributed to being turned over by ubiquitination. The crossover of BIN2 from the BR network to other signaling pathways was also observed. The auxin transporter PIN3 was identified through PL–MS, explaining the reason that BR modulates polar auxin transport [68,69]. Phototropism-related genes, phototropin 1 (PHOT1), nonphototropic hypocotyl 3 (NPH3), and an NPH3 family protein were also found. The findings suggest how BR mediates plant development while mitigating various other pathways through the negative regulatory factor BIN2.

### 3.2. Single-Molecule Technologies for Studying BR System

Single-molecule methods facilitate the observation of the dynamic interaction processes of target molecules. Compared with the aforementioned conventional techniques, which can identify new components of signaling but provide limited quantitative and dynamic data, single-molecule methods study biological macromolecules one-by-one in real-time and provide valuable insights into the fundamental biochemical and biophysical properties of the target biological system. However, only a few studies have used these methods to examine BR signaling transduction. For example, Wang et al. used variable-angle total internal reflection fluorescence microscopy (VA-TIRFM) and fluorescence correlation spectroscopy/fluorescence cross-correlation spectroscopy (FCS/FCCS) [70,71], Song et al. used colocalization single-molecule spectroscopy (CoSMoS) based on total internal reflection fluorescence microscopy (TIRFM) [72,73], and Hink et al. used the photon counting histogram (PCH) model and FCCS [71,74]. These studies have provided unique information on the BR system and demonstrated how single-molecule methods are powerful and promising tools for studying fundamental questions in plant science.

#### 3.2.1. TIRFM and VA-TIRFM

**Figure 3:**
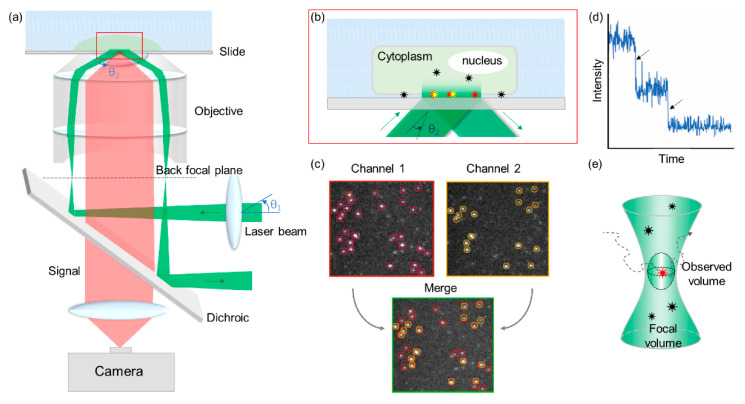
(**a**) Schematic of the total internal reflection fluorescence microscopy (TIRFM) and variable-angle (VA)-TIRFM layout. A focused laser beam (green) is reflected at the back of the objective focal plane, passed through the objective lens, and totally internally reflected. The signal (red) passes through the dichroic and is captured on a camera. By changing the angle of the laser beam (θ_1_), the incident angle (θ_2_) is variable and converts TIRFM to VA-TIRFM. (**b**) Enlarged view of the total reflection area in the sample and slide surface. Fluorophores within the evanescent filed field (approximately 100 nm) were excited (red and yellow). (**c**) Schematic representation of CoSMoS. If fluorophore-s labeled target proteins associate with each other, positions of spots in each channel (red box in channel 1 and yellow circle in channel 2) can be overlaid in the merged image. (**d**) An example of the double-step bleaching event trajectory. (**e**) Schematic representation of fluorescence correlation spectroscopy/fluorescence cross-correlation spectroscopy (FCS/FCCS) and photon counting histogram (PCH). The diffusion of fluorophore-labeled molecules in and out of the observed volume induces fluctuations in fluorescence with time. Their diffusion parameters and number of particles can be obtained by fitting the fluorescence fluctuation curve to an adequate diffusion model.

TIRFM images are produced by illuminating the sample with an evanescent wave, which is generated when the incident light is totally reflected at the interface between the slide and the specimen (Figure 3a,b) [72]. By using selectively excited fluorophores within the evanescent field (approximately 100 nm), TIRFM can reduce background noises and thus generate high-contrast images while minimizing cellular photodamage.

TIRFM is uniquely suited to visualize processes that occur in and around the membrane of living cells. However, plant cells have a rigid cell wall surrounding the plasma membrane, which restricts the penetration of the evanescent field and scatters the illumination. In 2000, Rohrbach developed a multiangle evanescent-wave microscope, referred to as VA-TIRFM, which overcame this shortcoming [75]. By controlling the variation of the incident angle, an evanescent wave can penetrate as deeply as 300 nm, enabling the observation of the deeper areas.

#### 3.2.2. FRET

FRET is a popular tool to measure the dynamic activities of biological molecules at the nanoscale [76,77]. In FRET, a donor fluorophore absorbs a photon and nonradiatively transfers this energy to an acceptor fluorophore when the distance separating them is less than 10 nm. The efficiency of FRET varies in proportion to the inverse sixth power of the distance between the donor and acceptor molecules. Therefore, measuring the FRET efficiency can enable the calculation of the precise donor–acceptor distance, which reflect labeled protein–protein or protein–ligand distances, at the angstrom level. By real-time tracking of nanoscale conformational changes, protein–protein interaction events, or ligand binding events at the single-molecule level, the molecular mechanism of macromolecules can be revealed [78]. Recently, single-molecule FRET has been widely used to study conformational changes of biological molecules and intermolecular interactions. However, only a few works have used FRET in plant systems. Compared with BiFC, which only uses one fluorophore color, the background fluorescence from chloroplast precludes the selection of proper FRET donor–acceptor pairs whose spectra must be separated from the background simultaneously [79]. This challenge has been overcome by combining single-molecule FRET with fluorescence lifetime imaging microscopy [80], where the contrast is based on the lifetime of individual fluorophores rather than their emission spectra. This has led to the exploration of numerous interactions (e.g., BRI1–SERK3/BAK1 and BRI1–P-ATPase interactions) with bulk, but not single-molecule, FRET measurements [81,82,83].

#### 3.2.3. CoSMoS

CoSMoS is a powerful method to investigate biomolecule reaction mechanisms of multiple components, including assembly pathways [73], compositional heterogeneity, and kinetics. When a complex is formed, the emission from fluorophore-labeled biomolecules colocalizes to the same location (Figure 3c). By real-time imaging localization of target molecules, CoSMoS can simultaneously observe interaction dynamics and correlate processes along the reaction pathway. However, due to the diffraction limit, CoSMoS is unsuitable for observing molecules at high concentrations.

During single-molecule experiments, fluorophores usually photobleach (become nonfluorescent due to photodamage) in seconds and exhibit a stepwise intensity trajectory (Figure 3d). Photobleaching can be postponed by reducing the light intensity or scavenging the oxygen surrounding the fluorophores. The number of stepwise drops in each spot can be interpreted as the number of the molecules. Therefore, stepwise photobleaching can be used in determining the stoichiometry or analyzing oligomerization states [84,85,86].

#### 3.2.4. Fluorescence Correlation Spectroscopy/Fluorescence Cross-Correlation Spectroscopy (FCS/FCCS) and Photon Counting Histogram (PCH)

FCS and PCH with a confocal microscope are techniques in which spontaneous fluorescence intensity fluctuations are measured in a microscopic detection volume of approximately 10^−15^ L defined by a tightly focused laser beam (Figure 3e) [74,87]. When a small number of molecules diffuse through this exceedingly small detection volume, molecules with smaller weight diffuse more rapidly than large molecules and result in a shorter fluorescence burst duration. These diffusion parameters can be obtained by analyzing the time-dependent decay of fluorescence fluctuations (FCS) or the amplitude distribution of the fluorescence bursts (PCH) [88].

To study direct interaction or colocalization of two types of particles, dual-color FCCS was developed [71]. In dual-color FCCS, fluctuating fluorescence signals from two target proteins labeled in different colors are detected in each channel. FCCS can detect crosstalk and measure reaction rates, colocalization dynamics, fractions of reacting molecules, and complex mobility [89].

#### 3.2.5. Discoveries in BR Signaling Pathway by Using SM Methods

Hink et al. were the first to apply PCH and FCCS on BR system in live plant cells to study the homodimerization of CFP/YFP-labeled *Arabidopsis* receptor proteins [88]. They found that both AtSERK1 and BRI1 in the plasma membrane can form homodimers, but no oligomeric structures were detected for AtSERK3.

By applying VA-TIRFM and FCCS, the membrane dynamics of GFP-labeled BRI1 was observed. Wang et al. reported that (1) BRI1-GFP particles exhibited multiple diffusion types in the membrane; (2) BRI1 endocytosis is clathrin-dependent and regulated by BR; and (3) membrane microdomains contribute to BRI1 partitioning and endocytosis and even affect BR signaling. By counting the photobleaching steps of BRI1-GFP, BRI1 molecules on the membrane were found to function as monomers or dimers [90].

Song et al. were the first to use CoSMoS based on a multicolor TIRFM and a homemade flow cell to perform an in vitro investigation of the signaling timescales of the major BR signaling components BES1 and BIN2 of *A. thaliana* [86,91]. By precisely controlling the reaction conditions (such as ATP and oxygen concentrations), the association and dissociation of BIN2 with BES1 were controlled and observed in real time. They found that the reactive oxygen species directly regulate the activity of BIN2 and thus control the interaction between BIN2 and BES1. Furthermore, ATP-related rate constants of BIN2 associating with ATP and phosphorylating BES1 were determined. By counting the photobleaching steps, BES1 and 14-3-3κ were proved as working in monomer and dimer, respectively [86,91].

Compared with findings that H_2_O_2_ induces the oxidative modification of BZR1 by traditional biological methods [37], single-molecule experiments enable more direct and real-time proofs for controllable participants in a pure environment. Song et al. have provided a platform for studying the dynamic interactions of signaling components that respond to internal or external cues, which is challenging to be discovered by conventional physiological and biochemical methods. Taken together, these studies emphasize that the single-molecule technique is a powerful and promising tool for studying fundamental questions in plant science.

## 4. Concluding Remarks

Various methodologies can be applied to facilitate the identification of new BR members and foster our comprehension of BR signaling and other signaling networks. In this review, we introduce several recent developments of technologies and illustrate how they contribute to our current understanding of the phytohormone BR. The method used in BR signaling is also available for other signaling pathways, particularly PL and single-molecule technologies. Notably, not many applications of these novel methodologies exist in the field of BR research, indicating that the usage of these methods is still at its budding stage, and many opportunities await. We believe that these methods will develop into powerful plant science research tools.

In addition, there are still many unanswered questions in BR signaling pathway research such as how the tissue specific development is regulated and whether non-coding RNA functions in BR signaling regulation. Besides phosphorylation and acetylation, does another modification exist and how does it regulate the BR signal? How do the redundant genes work? How does the BR signal affect modifications of the whole plants? Therefore, we still need to develop new methods for different conditions in BR signaling research, such as tissue specific signal-cell sequencing, next generation sequencing, and proteomics of post-translational modifications. Moreover, almost all of these methods are based on bioinformatics. Furthermore, comparative studies of BR signal pathway across different plant species will lead to a better understanding of the evolution and better application of BR signal pathway.

## Figures and Tables

**Table 1:** Proximity labeling (PL) methods categorized by the enzyme used.

Method	Applications	Remarks	Tag	Inducer	Reaction Time	Toxicity	References
Peroxidase based							
EMARS	Horseradish peroxidase (HRP)-based method: reactions at the cell surface	Working distance up to 300 nm; works on cell surfaces	Biotin or fluorescein	H_2_O_2_ with arylazide biotin or fluorescein arylazide	15 min	Free radicals	[49,50]
SPPLAT			Biotin	Tyramide-biotin and H*2*O*2*	5 min	Free radicals	[51]
APEX; APEX2	Engineered ascorbate peroxidase; proteomics of a subcellular compartment	From a plant ascorbate peroxidase; does not provide a history of protein associations	Biotin; targeting tyrosine, tryptophan, histidine, and cysteine	Induced by H_2_O_2_ under biotin-phenol additives	1 min	Free radicals	[53,54]
Binary-candidate method							
BirA/BAP	Binary-candidate method: modify both bait and prey proteins, can be applied across cells	BirA has a bacterial origin and is therefore orthogonal to mammalian or plant cells	Biotin	Biotin + ATP		Low	[46]
BLINC/ID-PRIME		Detection by streptavidin linked fluorophores staining	Biotin or lipoic acid (LA)	ATP with biotin or LA	2–15 min	Low	[47]
SPARK; SPARK2		Luciferase fused LOV domain; BRET type mechanism	Reporter gene	Blue light or luciferin	8 h	Photo-toxicity	[48]
Promiscuous PL enzyme							
BioID; BioID2	Promiscuous biotin ligase fused to a bait protein	Mutated from BirA; works within 10 nm; used ~37 °C	Biotin; target amines (including Lys)	Biotin supplementation	~16 h in plants	Low	[55,56]
TurboID		Improved from BioID; works at room temperature and above	Biotin; target amines	Biotin supplementation	~10 min	Low	[57]
PUP-IT (pupylation-based interaction tagging)	For identifying membrane protein interactions; bacterial Pup conjugation system	pafA, a gene encodes Pup ligase	Pup (conjugate to Lys)	Doxycycline to induce Pup(E) expression; in the extracellular format, PafA can be engineered to FRB and induce by rapamycin	24 h	Low	[58]
NEDDylator	From the NEDD8 pathway in mammalian cells	Done in vitro at ~37 °C	NEDD8 (conjugate to Lys)	HB-NEDD8	2 h	Unknown	[59]
EXCELL	For marking cell–cell interactions	Mutated from SrtA to recognize monoglycine at N-terminal promiscuously	LPXTG pentapeptide	Condition depending on cell line and transfection rate		Low	[60]

## Abbreviations

2D-DIGE	Two-dimensional difference gel electrophoresis
AD	activation domain
APEX	an engineered ascorbate peroxidase
BAK1	BRI1-associated kinase 1
BAP	biotin acceptor peptide
BD	binding domain
BEH1-4	BES1/BZR1 homolog 1-4
BES1	BRI1 ethyl methanesulfonate suppressor 1
BES1-h	The BES1 hextuple mutants
BiFC	bimolecular fluorescence complementation
BIN2	BR-insensitive 2
BioID	biotin identification
BirA*	a BirA mutant
BKI1	BRI1 kinase inhibitor 1
BLINC	biotin labeling of intercellular contacts
BRI1	BR-insensitive 1
BRs	Brassinosteroids
BRZ	brassinazole
BSKs	BR signaling kinases
BSLs	BSU1 like
BSU1	BRI1-suppressor 1
BZR1	brassinazole resistant 1
*bzr1-1D*	*bzr1-1 Dominant*
CDG1	constitutive differential growth 1
CoSMoS	colocalization single-molecule spectroscopy
CRISPR	clustered regularly interspaced short palindromic repeats
DSBs	double-strand breaks
EMARS	enzyme-mediated activation of radical source
EMS	ethyl methanesulfonate
FCS/FCCS	fluorescence correlation spectroscopy/fluorescence cross-correlation spectroscopy
FRET	Förster-type resonance energy transfer
gRNA	guide RNA
HRP	horseradish peroxidase
ID-PRIME	interaction-dependent probe incorporation mediated by enzymes
LA	lipoic acid
LC–MS/MS	liquid chromatography–tandem mass spectrometry
MAKR1	Membrane-associated kinase regulator
MS	mass spectrometry
NLRs	nucleotide-binding leucine-rich repeats
NPH3	nonphototropic hypocotyl 3
PAGE	Polyacrylamide gel electrophoresis
PCH	photon counting histogram
PHOT1	Phototropin 1
PL	proximity labeling
POI	protein of interest
PP2A	protein phosphatase 2A
PPIs	protein–protein interactions
PUP-IT	pupylation-based interaction tagging
RNP	ribonucleoprotein
SnRK2.2/2.3/2.6	Snf1-related Kinase 2.2/2.3/2.6
SPARK	specific protein association toll giving transcriptional readout with rapid kinetics
SPPLAT	selective proteomic PL using tyramide
T-DNA	Transfer DNA
TIR	toll/interleukin-1 receptor
TIRFM	total internal reflection fluorescence microscopy
TMV	tobacco mosaic virus
TTL	transthyretin-like protein
UBR7	a putative E3 ubiquitin ligase
VA-TIRFM	variable-angle total internal reflection fluorescence microscopy
Y2H	yeast two-hybrid
